# When Action Meets Emotions: How Facial Displays of Emotion Influence Goal-Related Behavior

**DOI:** 10.1371/journal.pone.0013126

**Published:** 2010-10-01

**Authors:** Francesca Ferri, Ivilin Peev Stoianov, Claudia Gianelli, Luigi D'Amico, Anna M. Borghi, Vittorio Gallese

**Affiliations:** 1 Department of Neuroscience, University of Parma, Parma, Italy; 2 Department of General Psychology, University of Padua, Padua, Italy; 3 Department of Psychology, University of Bologna, Bologna, Italy; 4 Institute of Psychiatry, University of Chieti-Pescara, Chieti, Italy; 5 Institute of Cognitive Sciences and Technologies, National Research Council, Rome, Italy; 6 Brain Center for Motor and Social Cognition, Italian Institute of Technology, Parma, Italy; University of Minnesota, United States of America

## Abstract

Many authors have proposed that facial expressions, by conveying emotional states of the person we are interacting with, influence the interaction behavior. We aimed at verifying how specific the effect is of the facial expressions of emotions of an individual (both their *valence* and *relevance/specificity* for the purpose of the action) with respect to how the action aimed at the same individual is executed. In addition, we investigated whether and how the effects of emotions on action execution are modulated by participants' empathic attitudes. We used a kinematic approach to analyze the simulation of feeding others, which consisted of recording the “feeding trajectory” by using a computer mouse. Actors could express different highly arousing emotions, namely happiness, disgust, anger, or a neutral expression. Response time was sensitive to the interaction between *valence* and *relevance/specificity* of emotion: disgust caused faster response. In addition, happiness induced slower feeding time and longer time to peak velocity, but only in blocks where it alternated with expressions of disgust. The kinematic profiles described how the effect of the specificity of the emotional context for feeding, namely a modulation of accuracy requirements, occurs. An early acceleration in kinematic relative-to-neutral feeding profiles occurred when actors expressed positive emotions (happiness) in blocks with specific-to-feeding negative emotions (disgust). On the other hand, the end-part of the action was slower when feeding happy with respect to neutral faces, confirming the increase of accuracy requirements and motor control. These kinematic effects were modulated by participants' empathic attitudes. In conclusion, the social dimension of emotions, that is, their ability to modulate others' action planning/execution, strictly depends on their *relevance* and *specificity* to the purpose of the action. This finding argues against a strict distinction between social and nonsocial emotions.

## Introduction

Facial expressions are widely acknowledged as essential to express emotional states. A long tradition [1]–[3] assumes them as configurations of muscles conveying the inner emotional state of a person. Recent studies clearly suggest the relevance of these physical components for emotion understanding and thus empathy. A consistent body of evidence shows that iconic symbols of emotions, like pictures portraying people showing anger or disgust, are powerful enough to evoke a congruent activation of the facial muscles of the observer in line with the idea of embodied simulation [4]–[7]. A different perspective [8]–[11] underlines the role of emotions as social tools conveying behavioral intentions or action requests during social interactions. Summarizing, emotional processing implies different dimensions, such as valence, arousal, approach/withdrawal, and sociality. Indeed, different emotions have been proposed to be characterized by various degree of sociality: for example disgust is considered a non social emotion [12], [13].

Physical and social aspects of facial expressions have been addressed separately, so far. However, successful social interactions require both the ability to detect others' emotions and to adapt our behavior to theirs. Our work moves from the assumption that in processing of emotions the physical and the social dimensions are strictly interrelated. A given facial expression is both the manifestation of an internal state and a social tool, as it influences others' actions. This is in line with the approach proposed by embodied theories of emotions [4], [5], according to which experiencing and understanding emotions share the same sensorimotor and visceromotor systems (see also [14]–[16]).

Only few studies have investigated the relationship between emotions and action planning/execution. Some studies employing visual stimuli demonstrated that emotions facilitate actions (e.g., [17]). For example, Oliveri et al. [18] showed in a TMS study that visual cues with negative emotional content (i.e., disgusting) increase the excitability of the motor cortex. Other studies have demonstrated that also linguistic stimuli of positive and negative valence can trigger approach and avoidance behaviors, respectively [19]–[23]. In a different framework, recent findings showed that motor behavior is also modulated by the social situation of interaction with a conspecific [24], [25]. Even though all these lines of research can be relevant to our approach, to the best of our knowledge only one study specifically investigated the effects of facial expressions on motor behavior. Indeed, Seidel et al. [26] have recently performed a well-controlled study of motor tendencies (approach and avoidance) in response to happy, sad, angry, and disgusted faces.

Differently from what done by Seidel et al. [26], we focused on the effects of facial expressions on a motor behavior, which is intrinsically emotional and intimate, such as feeding. We chose such a situation on the basis of different reasons. First, we relied on a previous study by Ferri et al. [27] showing that the kinematics of the feeding action is affected by the intention to feed others, rather than oneself, and by the “sociality” of the context. Second, we were interested in a motor task providing us the possibility to contrast the effect of an implicit processing of emotional information tuned with the particular motor behavior, with the effect of less specific emotional information. Consider the difference between anger and disgust with respect to feeding: a baby could express anger when she is hungry, therefore anger can be relevant to the feeding behavior. However, there is no specific association between the expression of anger and the presence of a food. Angry expressions generally communicate the request to go away [26], [28], [29]. Thus, angry expression in the presence of a food can be relevant for the feeding behavior, because it can inhibit it, but it is not specific. Conversely, from an evolutionary point of view, disgust signals a request to avoid, e.g. the food just consumed or noxious stimuli. However, the expression of disgust in presence of a piece of food is commonly interpreted as associated with the desire of rejecting that food. Repacholi et al. [30] demonstrated by using a food-request procedure that 18-month-old children are able to understand food-related others' desires. Children correctly inferred that the experimenter wanted the food associated with her prior happy expression, whereas she did not want the food associated with her previous expression of disgust.

In our study we tested whether and how the kinematics of an action targeting another individual is modulated by both affective and social content of emotions expressed by the same individual; the former referring to the dimension of valence (positive/negative) and the latter referring to the relevance and specificity of the particular emotion to the interaction.

Indeed, we did not follow any assumed distinction between social and non-social emotions, such as the one proposed by Britton et al. [12], on the one hand, and by Adolphs, Baron-Cohen, & Tranel [13], on the other. We moved from the hypothesis that any kind of emotion might acquire social relevance in a specific context of interaction. Consider the case of disgust: eminently categorized as a non-social emotion [12], it might acquire a social role in the specific situation of feeding a conspecific.

We tested this hypothesis by using a kinematic approach to study the behavior of participants simulating feeding. Simulating feeding might seem quite unnatural. Note, however, that feeding behavior occurs rather frequently not only with kids, but also with ill people and in romantic situations. Thus, feeding can be considered as one as the most familiar behavior, between those intrinsically emotional and intimate. Static pictures portraying faces of actors expressing positive, negative or neutral emotions were presented on a screen. After a delay, a piece of food appeared at the bottom of the screen and participants had to simulate to “grasp” the food, and to “bring it to the mouth” of the actor by using a mouse. In half of the blocks the negative emotion was relevant and specific for the feeding action (disgust, *relevant blocks*), in the other half it was not specific (anger, *irrelevant blocks*).

We defined negative emotions and experimental blocks by using the term “relevance” to intend both relevance and specificity for the task, as clarified earlier. Note that the notion of “relevance to the task” is widely used in the literature on attention, also when applied to studies on emotions, as testified by findings of an interaction between emotional and attentional functions [31].

The blocked design allowed us to study together the role of both the affective (valence) and the social content of the emotions. Importantly, any effect of the emotional context on the feeding action would be implicit, as participants always had to perform it.

Our prediction was that the observation of emotions would differently affect the kinematics of an action depending on whether the emotions are relevant or not to the purpose of the interaction (here, feeding someone). More specifically, we focused on the analysis of temporal measures.

We expected an emotional modulation of both the early “grasping” and the following “bringing to the mouth” phases of the feeding action. Indeed, we investigated early effects, mainly function of the intrinsic nature of emotions (valence, intensity), by measuring response time. In addition, we measured feeding time and time to peak velocity, in order to study late effects. The former measure would reflect the effect of emotional expressions on more automatic processes; the latter, on more controlled processes [26].

Preceding studies (e.g., [32], [33]) questioned the assumed automatic association [29], [34] between the valence of facial expressions and action tendency. However, there are contradictory results in the literature related to the effect of emotions on social behavior (approach and avoidance). Concerning anger emotion, Marsh et al. [28] showed that angry faces facilitated avoidance behavior; whereas, Wilkowski et al. [35] demonstrated that angry facial expressions potentiate approach-motivated motor behaviors. Similarly, concerning behavioral reactions to expressions of disgust, Seidel et al. [26] found unclear behavioral tendencies. Indeed, disgust elicited withdrawal in a rating task, whereas no significant tendencies emerged in the joystick task, which is a task revealing approach (pulling a joystick towards themselves) and avoidance (pushing it away from themselves) tendencies. Conversely, there is much consensus on the effect of happy faces in communicating an invitation to cooperate [26], [29].

Given these contradictory data, we could not predict a specific direction of the results (facilitation, interference), at least regarding behavioral tendency evoked by angry and disgusted faces. However, we advanced the following two hypotheses. First, response time and kinematics of the mouse path should be modulated by context-specific face processing [36]. More precisely, they should depend on the specificity of the emotional context of each block for feeding action. Second, a tendency to approaching and being more accurate should be observed with happy faces. To verify the second hypothesis, we performed also analysis of kinematic profiles, which would describe the effects of emotions on movement dynamics. In fact, kinematic aspects of movements are informative about the way a social interaction is performed. For example, Ferri et al. [27] showed that participants were slower and more accurate when feeding others, rather than feeding oneself or placing the food into a mouth-like aperture in a human body shape. We specifically focused on kinematic profiles related to happiness, because it is the emotion most likely associated with an approaching behavior [26], [29].

Finally, we also predicted that these kinematic late effects would be modulated by participants' empathic attitudes.

## Methods

### Participants

Thirty-four students (16 women; mean age  = 27,5) from the University of Chieti participated in the experiment. All were right handed (Edinburgh Handedness Inventory score >0.85) and had normal or corrected-to-normal vision (correction <0.75). All were naive as to the purposes of the experiment and gave their written informed consent. The experimental protocol was approved by the Ethics Committee of the “G. d'Annunzio” University, Chieti.

### Apparatus and stimuli

Participants sat 60 cm from the computer screen, with their right hand placed over a mouse (Microsoft Wireless Notebook Laser Mouse 7000), positioned on a table in correspondence with the midline of the computer screen (starting position). The action of feeding was simulated by “attaching” the food image to the mouse: participants “grasped” the food by a left button-click and brought it to the actor's mouth by dragging the mouse. The use of a computer mouse-tracking method for recording participants' hand motion has been already reported in previous studies (e.g., [22], [23], [37]).

**Figure 1:**
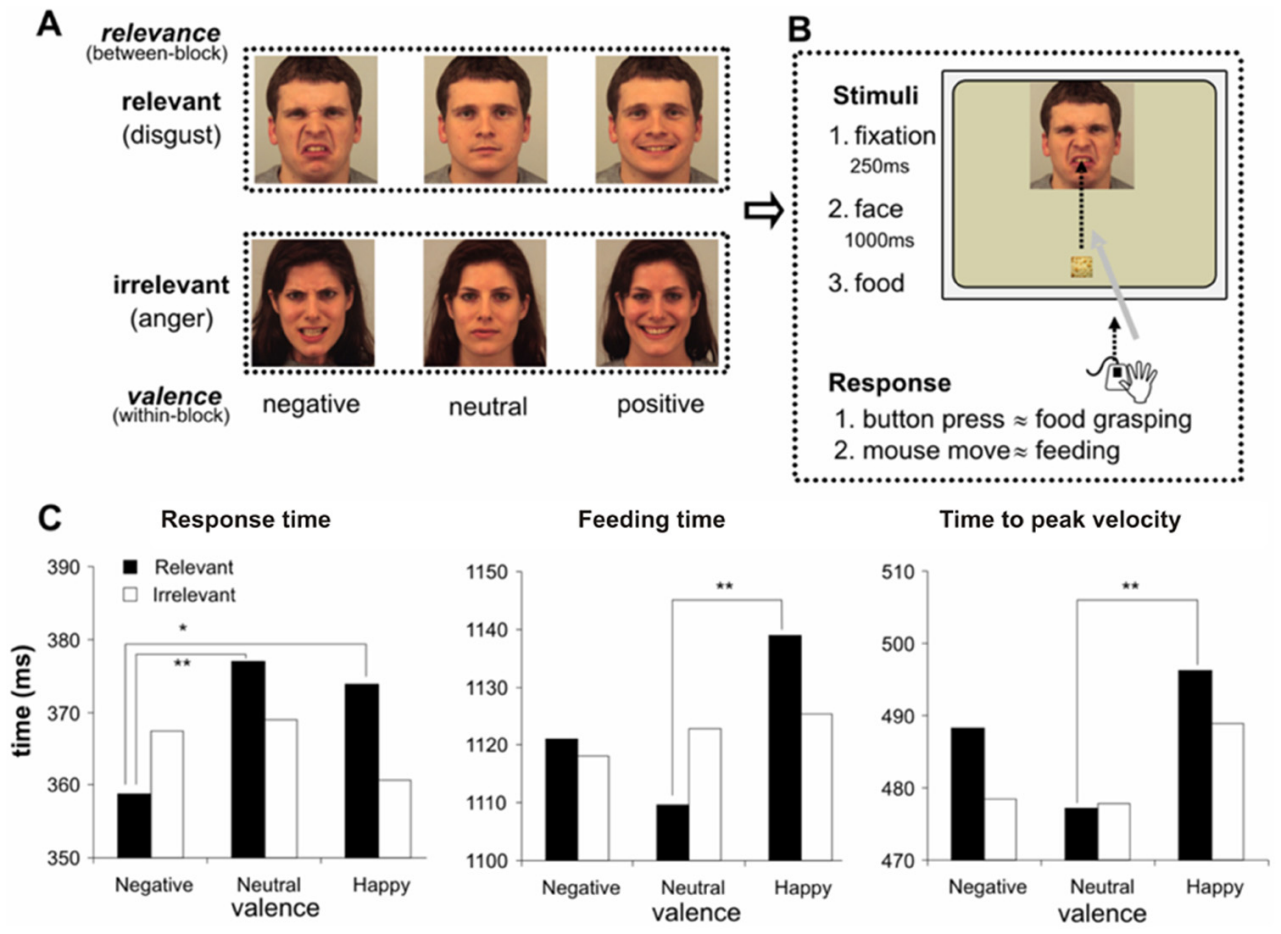
Method and timing results. (**A**) Experimental design. Emotional *valence* (negative, neutral, positive) is a within-block factor. *Relevance* of the negative emotion to the feeding action (relevant: disgust, irrelevant: anger) was a between-block factor. Face pictures in figure 1A are examples of stimuli selected from KDEF database and used for the standardized presentation of the emotional expressions. (**B**) Procedure. Participants were presented with a facial stimulus (1000 ms) following brief fixation; as soon as a food-image appeared (GO-signal) they had to “*grasp*” it (mouse button-click) and “*feed*” the actor. (**C**) Results. Grasping-time, feeding-time, and time-to-peak velocity were modulated by both *valence* and *relevance*: “grasping” was faster only in the relevant negative condition (disgust) and feeding was slower only in the relevant positive condition.

The Karolinska Directed Emotional Faces database (KDEF; ref. [38]) was used for the standardized presentation of the emotional expressions. Four male (KDEF index: 08, 23, 25, 31) and four female actors (KDEF index: 07, 13, 20, 20) expressing either disgust or anger, happiness, or displaying a neutral expression were selected on the basis of the hit rate accuracy scores. The average scores per emotion for the selected actors were 0.74±0.13 (disgust), 0.72±0.15 (anger), 0.91± 0.09 (happiness), and 0.64±0.29 (neutral), (0 = never correctly identified; 1 = always correctly identified). Each face, sized 535-by-561 pixels, was presented with the centre of the mouth always horizontally centered and located 54 pixels below the centre of the screen. Twelve pictures of food (e.g., biscuit, cracker, pastry, small pizza), balanced for taste (sweet, neutral, salty) and shape (round or square), were presented with a transparent background below the face (265 pixels apart from the mouth centre). Food pictures sized 81-by-81 pixels were horizontally centered, with up to 10 pixels displacement, to avoid kinematic habituation (Figure 1A). Stimuli were displayed using Eprime software [39], [40]. Importantly, all the twelve different food items were presented in a random order, so that each food item was associated only once with a given actor. This random order was different for each actor and experimental subject. Therefore, no eventual coherence of a specific emotion and a piece of food could explain the results.

### Simulation of feeding procedure

Each trial started with the participant holding the mouse with the index finger on the left key and a black fixation cross appearing at the centre of the white screen for 250 ms. A face picture followed. After a delay of 900–1100 ms a food-image (GO-signal) was presented (Figure 1B). Participants were explicitly instructed to simulate a feeding behavior. Indeed, they were told: “In this experiment you will simulate to feed a person. Her/his face picture will appear in the centre of screen. As soon as a piece of food will appear below the face, grasp it by clicking the mouse button and feed the person. To bring the piece of food to her/his mouth, you will have to drag the mouse up to the centre of the mouth”. The trial ended as soon as participant reached the actor's mouth (up to 5000 ms) (Figure 1B). During the subsequent 4200–4800 ms ITI, participants had to place the mouse back on the starting position.

The experimental session contained 8 blocks, one for each of the actors. A block consisted of 12 trials, in which the actor could express a *positive*, a *neutral*, or a *negative* emotion. Each emotion was repeated four times (Figure 1A). The positive emotion was happiness. In half of the experimental blocks the negative emotion was relevant and specific to the feeding action (disgust, *relevant blocks*); in the others it was not specific (anger, *irrelevant blocks*).

The assignment of the negative emotion was gender-balanced. Actors (between-block) and the emotional valence they expressed (within-block) were shown in random order. The experimental session was preceded by a training session with two additional actors. In total, there were 12 practice and 96 experimental trials.

### Measures of trait empathy

Participants were asked to complete the Italian version [41] of the Interpersonal Reactivity Index (IRI; ref. [42]–[44]). IRI is a 28-item self-report survey that consists of four subscales: Empathic Concern (EC) and Personal Distress (PD) subscales, referring to more genuine emotional aspects of empathy, whereas Perspective taking (PT) and Fantasy scale (FS), assessing more cognitive aspects of empathy.

### Rating of expressions

To be certain that participants' evaluation of emotions were in line with the hit scores of the selected stimuli, after the experimental session a randomly selected subset of twenty participants was presented with all the expressions and asked i) to qualify each image as angry, disgusted, neutral or happy; ii) to rate the expression on a 5-level scale. Pictures were shown on a computer screen in pseudo-random order.

### Data Recording and Analysis

We recorded the *response time* and the mouse path, here referred to as “feeding trajectory”, sampled at 75 Hz (every screen refresh). Mouse trajectories were reconstructed by filtering all the sampled mouse positions with an 8-point moving-average filter. Similarly, velocity and acceleration were calculated for each point of the feeding trajectory. In addition, we calculated the value of peak velocity on the correspondent profile, and consequently detected onset and offset of a discontinuous motion with velocity greater than 1% peak-velocity threshold.

*Feeding time* was the time between the onset and the offset of the feeding movement. *Time to peak velocity* was the time to reach peak velocity from motion onset.

Fifty-point time-normalized *velocity* and *acceleration* average *profiles* were calculated for each subject and each condition (*absolute profiles*) by means of interpolating the original profiles and sampling them at fifty equidistant time-points ranging from motion onset to offset. *Relative-to-neutral profiles* were calculated as point-by-point differences between either velocity or acceleration profiles of non-neutral conditions (*happy*, *disgust*, *anger*) and the neutral profile presented in the same block *(relevant/irrelevant*). All means were cleaned of 2.5-std outliers.

ANOVAs were carried out on the mean values of the participants' response time, feeding time and time to peak velocity. The within-subjects factors were emotion *valence* (positive, neutral, negative) and *relevance* of the negative emotion to the action (relevant, meaning relevant and specific; irrelevant, meaning not specific). In all analyses contrasts were two-tailed t-tests. The significance level was fixed at P<0.05. T-tests verified significant (p<0.05; FDR corrected, ref. [45]) per-point differences between the *absolute* profiles (non-neutral vs. neutral).

Finally, effects of the kinematic analysis were predicted with the measures of trait empathy by means of a linear regression analysis.

## Results

### Errors

Trials in which response times from the GO signal were faster than 150 ms (anticipations), slower than 800 ms (delayed responses), or followed mouse motion were considered as errors and discarded without replacement. Concerning the feeding movement, we excluded from the analysis trials in which feeding was completed in less than 700 ms (inaccurate mouse sampling), took longer than 3000 ms (too slow), or was not completed at all. “Grasping” anticipations occurred in 0.9% of the trials, delayed responses in 1.3%, and anticipated mouse motion in 0.8%. Feeding was too fast in 1.1% of the trials, too slow in 0.1% and did not reach the target in 3.2%. Thus, 7.4% of the trials were irregular and discarded.

Errors were not further analyzed since we focused on motion dynamics.

### Response time, Feeding time, Time to Peak Velocity

Response time was sensitive to the interaction between *relevance* and *valence* of emotion (F(66,2) = 3.2, MSe = 2250, p<0.05, *η* = 0.09). As predicted, relevant and specific negative facial expressions (disgust) differed from neutral and happy expressions (359±12.3 (1SE) ms vs. 377±14.0 (1SE) ms, and 374±13.7 (1SE) ms, respectively; p = 0.008 and p = 0.016). Anger, in quality of negative expression, could have caused an early effect, but did not modulate grasping execution because it was not specific for the task. Crucially, disgust, which was relevant and specific for the task, caused faster response time (Figure 1C)

As predicted, also Feeding time (Figure 1C) was sensitive to the interaction between *relevance* and *valence* (F(2,66) = 3.3, MSe = 3056, p<0.05, *η* = 0.09), although in a different way. Feeding happy actors was slower relative to the neutral condition, when the actors' negative expression in the same block was relevant and specific (1139±48.5 (1SE) ms and 1110±45.2 (1SE) ms, p<0.01), but not when it was not specific for feeding (1126±48.4 (1SE) ms and 1123±48.0 (1SE) ms; p = 0.79). This supports our prediction that context's relevance and specificity for the task are crucial in determining how carefully the feeding of happy faces is executed. Importantly, no significant difference was found between feeding disgusted and neutral actors.

Time to peak velocity was sensitive to *valence* (F(2,66) = 4.5, MSe = 3927, p<0.05, *η* = 0.12). Happy expressions delayed peak velocity relative to neutral ones (493±14.3 (1SE) ms and 477±13.6 (1SE) ms, p<0.01). Predicted contrasts, on the basis of the above feeding-time analysis, confirmed the interaction with *relevance*: the effect of *valence* holds for the relevant and specific (496±14.1 (1SE) ms vs 477±13.5 (1SE) ms; p = 0.007), but not for the not specific (488±15.4 (1SE) ms vs 478±14.5 (1SE) ms; p = 0.128) contextual negative emotion. This result is consistent with what found in the feeding time analysis.

Summarizing, these results show both a between- and a within-block effect of emotions on action execution: only in the *relevant blocks* the simulation of both grasping and feeding actions were specifically modulated. In particular, disgust caused a faster response time, while happiness increased the accuracy requirements, as it elicited a slower feeding action.

### Velocity and Acceleration profiles

**Figure 2:**
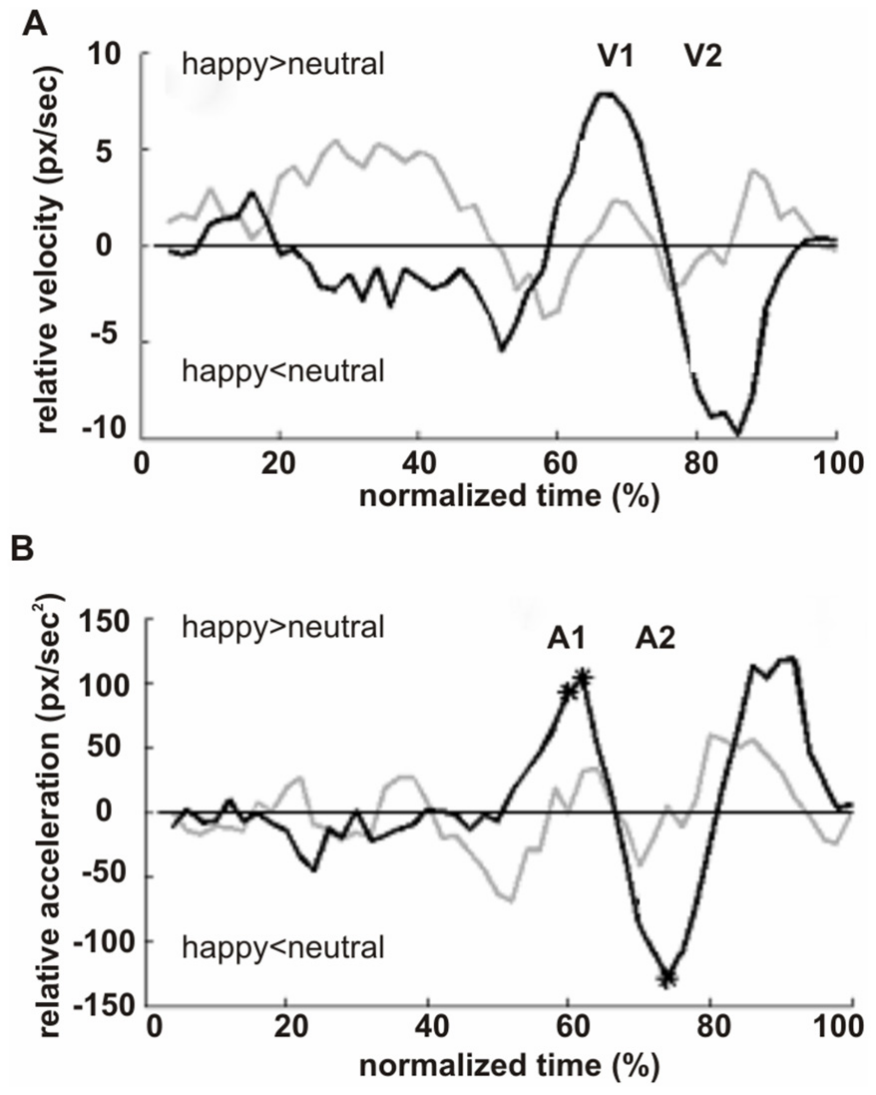
Velocity and acceleration profiles. Relative-to-neutral time-normalized profiles of velocity (A,) and acceleration (B) during feeding an actor expressing a positive emotion (i.e., happiness) in blocks with relevant and specific (disgust; black line) and not specific (anger; grey line) negative expressions. Stars indicate time-percentile intervals with significant differences between the *positive-expression* profiles compared to the corresponding *neutral-expression* profiles of the same block (p<0.05; FDR corrected).

The analysis of feeding time showed that in the case of relevant and specific emotional context, positive expressions delayed the participants' action execution with respect to neutral expression. To better characterize the dynamics of this effect, we focused on these trials analyzing the relative-to-neutral kinematic profiles of velocity (Figure 2A) and acceleration (Figure 2B) in *relevant* and *irrelevant blocks*.

We identified consistent kinematics differences between happy and neutral acceleration profiles only in the relevant and specific emotional blocks (black lines, Figure 2B), which we grouped in two phases. The first phase was at the beginning of motion deceleration (see *absolute profiles* in Figure S1), and comprised the 55–65 time-percentile interval of the acceleration profiles (indicated as A1 in Figure 2B). On the *relative acceleration profiles*, it corresponded to an average acceleration increase of 65.7 px/sec^2^ (i.e., 19.7%). As shown on Figure 2B, in that phase there were two time-percentile intervals in which the positive profiles significantly differed from the corresponding baseline intervals (p<0.05 per time-interval, FDR-correction). Within a short delay, this relative-to-neutral acceleration increase accumulated an average velocity increase of 5.2 px/sec (i.e., 1.6%) in the immediately following 60–75 time-percentile interval of the relative velocity profile (indicated as V1 in Figure 2A). Summarizing, an early acceleration of feeding behavior relative to the neutral condition occurred when actors expressed positive emotions (happiness) in blocks with relevant and specific negative emotions (disgust). Less deceleration in that phase, in which the hand is still away from the target, is an indicator of greater control on the movement.

The second phase characterized with consistent relative-to-neutral kinematics variations was also specific to the positive emotion in the *relevant blocks* and comprised the 70–80 time-percentile interval on the acceleration profiles (indicated as A2 in Figure 2B). On the *relative-to-neutral acceleration profiles*, it corresponded to an average acceleration decrease of 91.2 px/sec^2^ (i.e., 12.9%). As shown on Figure 2B, in that phase there was one time-percentile interval of the positive profile significantly different from the corresponding baseline (p<0.05, FDR correction). This relative acceleration drop accumulated an average velocity drop of 6.5 px/sec (i.e., 3.9%) in the immediately following 80–90 time-percentile interval of the relative velocity profile (indicated as V2 in Figure 2B). Thus, specifically in *relevant blocks*, the expression of happiness increased the accuracy requirement with respect to neutral face. This was obtained by means of a slower execution of the end-part of the feeding action.

In conclusion, these results showed that the deceleration phase of the feeding action was sensitive to the interaction between the relevance of the emotional context and the valence of the emotion expressed by the actor to be fed: feeding happy actors turned out to be the specific condition in which more control was required.

Finally, we asked whether our results depended on the inescapable fact that faces expressing different emotions had mouths of varying width. Indeed, according to the Fitts' law [46], larger objects are easier to detect and to target in pointing-like actions. Hence, they facilitate and speed-up movements. By calculating the number of pixels, we observed all actors' happy mouth to be greater than her/his neutral mouth (for details, see Figure S2). Thus, according to the Fitts' law, dragging time should be faster in case of happy expressions relative to neutral ones, which is the opposite of our finding. Our interpretation is that increased accuracy requirements in happy condition slowed down food-dragging, even though easier targets were presented.

### Effects of Personality

We investigated for potential predictive role of trait empathy on the specific effect in the velocity profiles (specific for expressions of happiness in *relevant blocks*) by means of linear regression analysis in which the average size of the relative velocity in the V1 and V2 periods were predicted by trait empathy measures. As predictors we considered only IRI-EC (assessing the tendency to experience feelings of sympathy and compassion for others in need) and IRI-PD (assessing the extent to which an individual feels distress as a result of witnessing another's emotional distress) scores, i.e. the two sub-scales more related to genuine emotional aspects of empathy, because of the focus of our work.

We decided to investigate the potential predictive role of trait empathy on velocity profiles, rather than on response time and feeding time, because we were interested in the social dimension of emotions. In other words, we aimed at investigating how emotions, as much as they were perceived by more or less empathic participants, affected the execution of a controlled interaction, rather than a more automatic reaction, even at a simulative level. In fact, kinematic aspects of movements are more informative about the way a social interaction is performed.

The regression model of the average velocity in the V1 period predicted 19.7% of its variance (F(2,30) = 3.7, p<0.05). Average velocity in V1 increased with IRI-PD (β = 0.37, t = 2.2, p<0.05), but IRI-EC was not a predictor of velocity in V1 (β = -0.21, t = 1.3, p = 0.22) (Table 1).

**Table 1:** Correlation between the average relative V1 and V2 velocity in the condition demanding greater accuracy, namely positive expression in relevant blocks, and subjective scores concerning two IRI subscales: Empathic Concern and Personal Distress.

*Relative velocity profiles*	V1 period		V2 period	
	*r*-value	*P*-value	*r*-value	*P*-value
*Measures of empathy*				
Empatic Concern	0.24	p = 0.22	0.44	p<0.05
Personal Distress	0.4	p<0.05	0.54	p<0.001

**Figure 3:**
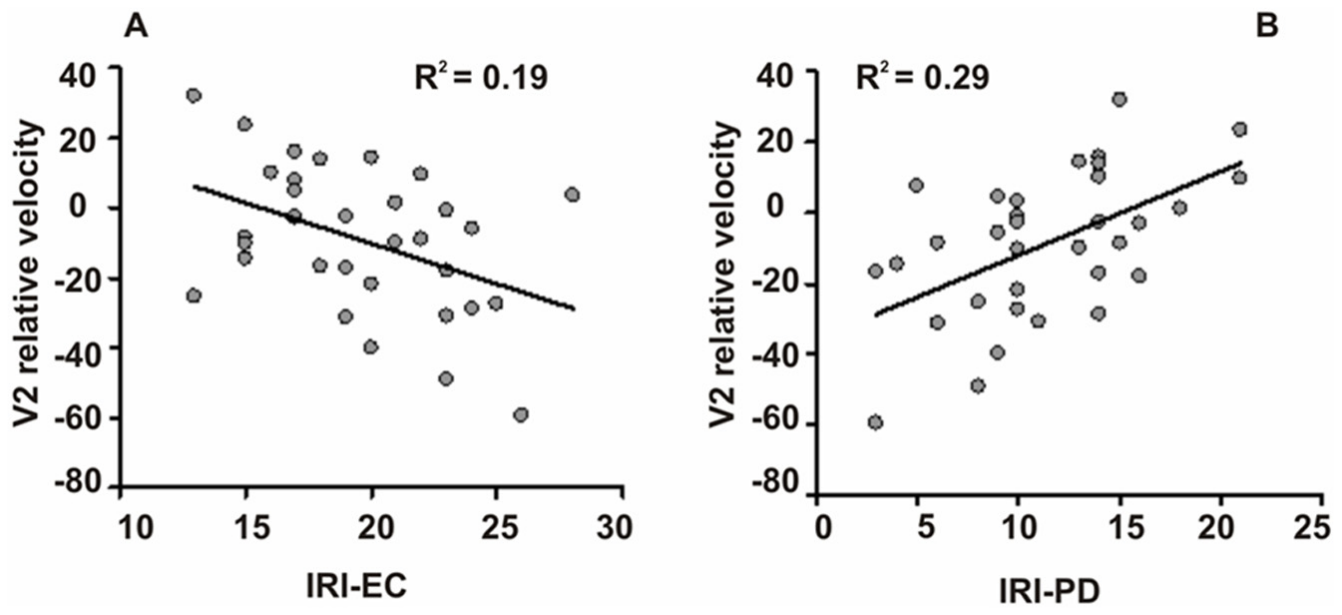
Effect of personality on velocity profiles. Per-subject average relative V2 velocity in the condition demanding greater accuracy, namely positive expression in relevant blocks, as a function of: (A) IRI-empathic concern and, (B) IRI- personal distress. R^2^ values refer to correlations with each of those predictors.

The regression model of the average velocity in the V2 period predicted as much as 42.5% of its variance (F(2,30) = 11.1, p<0.001). IRI-PD was again an important predictor similar to that in the V1 period: V2 velocity increased with the increase of IRI-PD (β = 0.49, t = 3.5, p<0.001), which corresponded to a lesser drop of velocity (Figure 3B, Table 1). Also IRI-EC was a significant predictor, whereby velocity decreased with higher IRI-EC scores, that is, the velocity drop increased (β = −0.38, t = 2.7, p<0.05; Figure 3A, Table 1).

Thus, the kinematic late effects were modulated by participants' empathic attitudes. The higher the EC score, the greater the motor control participants exercised in the final deceleration phase of the interaction. In addition, the more distressed by relevant expressions (disgust) were the participants, the greater the velocity. Hence, they showed weaker motor control in the entire deceleration phase.

### Rating the actor's expression

Happy expressions were the easiest to detect (0.6% error), followed by expressions of disgust (8.8% error), neutral (10.6% error) and anger (13.8%). Ratings followed a similar pattern: highest for happy (3.80), followed by disgust (3.57), anger (3.45) and neutral (3.43) expressions. Results are consistent with hit rate accuracy scores of the KDEF database. The two negative emotions did not significantly differ by error-rate and rating (p = 0.41; p = 0.55). Thus, we can rule out the possibility that the faster grasping response to disgusted, but not angry, actors, with respect to both happy and neutral faces, was due to a difference in their recognition.

## Discussion

The present study investigated the relationship between emotion perception and action planning/execution within a social context. More precisely, we studied: i) how specific (both in terms of valence and relevance) is the effect of the facial expressions of emotions of an individual with respect to how the action aimed at the same individual is executed; ii) whether and how the effects of emotions on action execution are modulated by participants' empathic attitudes. We used a kinematic approach to analyze the simulation of feeding others, which consisted of recording the “feeding trajectory” by using a computer mouse. Actors could express different highly arousing emotions, namely happiness, disgust, anger, or a neutral expression. In particular, we studied the effect on the action planning/execution of both the *valence* of emotions and of their *relevance/specificity*, for the purpose of the action.

Participants performed the experiment in a non-ecological situation. In fact, the experimental set up was explicitly designed to allow participants' simulation, by using static images, no feedback and no cooperation task. Indeed, our aim was to study the pure and specific effect of some basic emotions on a particular action: we wanted to avoid any confound between the effect of the social dimension of emotions, as they are capable to influence others' actions, and the effect of the sociality of a more complex experimental situation *per se*.

The analysis of response time showed early effects, depending on both the intrinsic nature of negative emotions and the dimension of sociality. Specifically, disgust caused faster “grasping”, being the only negative emotion relevant and specific for the feeding behavior. This result is in line with the data obtained by Oliveri et al. [18], demonstrating the increased excitability of the motor cortex triggered by disgusting scenes. It can be argued that, embodied-theories assumptions notwithstanding, perception of a picture of a prototypical expression of disgust is not an experience of disgust. However, a number of results seem to contrast this interpretation. For example, evidence from functional Magnetic Resonance Imaging (fMRI) studies showed that observing facial expressions of disgust and feeling disgust activated the same sites in the anterior insula [47]. Moreover, Jabbi et al. [48] showed that neural substrates involved in observing, imagining and experiencing disgust, such as anterior insula, are embedded in distinct functional circuits during the three modalities. This would suggest why observing, imagining and experiencing an emotion feels so different.

Finally, recent data obtained by Intracortical MicroStimulation (ICMS) experiments in rhesus monkey can help elucidating our observation that disgust is associated with faster motor response. Indeed, Caruana et al. [49] found that the stimulation of anterior insula induces behavioral reactions of discarding preferred food (e.g., spitting food when in the mouth and throwing it away when held in the hand, during ICMS).

Anger, in contrast, despite its features in common with disgust (both being negatively-valenced, highly arousing, withdrawal-related emotions), did not modulate the “grasping” simulation (response time), likely because it was not specific for the task. This demonstrates that since the very beginning of action preparation the dimension of sociality is crucial, cooperating with the dimension of valence and arousal in affecting action execution. This important role of the social dimension increases during the actual interaction, when approaching the actor to be fed. Indeed, happiness induced slower feeding time, but only in the *relevant blocks*, that is, when it alternated with expressions of disgust. Slower feeding time is consequence of longer time to peak velocity in the same condition, due to an increase of accuracy requirements. These findings are in accordance with previous reports of happy faces communicating an invitation to cooperate [29]. We do not intend to claim that we provided direct evidence on the specific effect of positive expressions. However, our results show that motor responses, here feeding simulation performed in specific-to-feeding emotional context, is more accurate when the displayed emotion induces caring for someone”, that is, higher accuracy [27]. In this respect, we extended our knowledge on the effect of specific-to-motor-behavior emotional cues on accuracy.

The kinematic profiles allowed us to study the effects of emotions on movement dynamics. This analysis not only confirmed the crucial role of social dimension on the late phases of action execution, but also provided a description of how this effect occurs. At the beginning of motion deceleration, an early acceleration in kinematic relative-to-neutral feeding profiles occurred when actors expressed positive emotions (happiness) in blocks with relevant and specific negative emotions (disgust). On the other hand, the end-part of the action was slower when feeding happy with respect to neutral faces, confirming the increase of accuracy requirements. The fact that the expression of happiness affected the feeding execution only when the emotional context of the whole block was relevant and specific, even if the same expression was presented also in the *irrelevant blocks*, is in line with the proposal of Aviezer et al. [50], of the “malleability” of emotion perception. They showed that the perception of basic facial expressions is context dependent. Similarly, in our study, where the context is a context of motor interaction, the emotional information is “read out” from the face according to the purpose of the interaction itself.

Importantly, these late kinematic effects were largely modulated by participants' empathic attitudes: as much as 42.5% of the velocity drop variance in the final approach towards the target was explained by Empathic Concern (the more empathic the subjects, the greater the velocity dip, i.e., greater motor control applied) and Personal Distress (the more the subjects are distressed by relevant and specific expressions, the smaller the velocity dip, i.e., the weaker the motor control). These results confirm the role of empathy in regulating social interactions and our ability to adapt our behavior to others' emotional state (e.g., [51]), as shown, for example, by the well-known literature on emotional contagion (e.g., [52]). Finally, the strong modulation of the kinematic effects by the participants' empathic attitudes supports our interpretation based on the embodied theories of emotions [4],[5]. Indeed, an alternative explanation of our findings could rely on a more cognitive hypothesis (see [37]), according to which mouse motion would be influenced by a conceptual categorization of the iconic symbols of emotions.

To summarize, we demonstrated that the social dimension of emotions, that is their ability to modulate others' action planning/execution, strictly depends on their relevance and specificity for the action: feeding a happy actor requires more accuracy compared to feeding a disgusted, but not an angry actor. This finding argues against a strict distinction between social and non social emotions.

In addition, the effect of relevance/specificity occurs very early, interacting with the effect of the other dimensions of valence and arousal, more intrinsically related to the nature of a given, specific emotion. In addition, our results corroborate the hypothesis that in processing of emotions the affective (arousal and valence) and the social dimensions are strictly interrelated, and that the social dimension plays an important role both very early, during action preparation, as well as later, during the effective interaction with others.

The present results open the possibility of investigating more thoroughly the interplay of action and emotion in specific social contexts. For example, by investigating other goal-directed actions with specifically related or unrelated emotions, one could expect the expression of anger to be more relevant and specific when the goal is to hurt someone. Further research is needed to investigate these fascinating issues.

## Supporting Information

Figure S1 Velocity and acceleration profiles. Absolute time-normalized profiles of velocity (A) and acceleration (B) during feeding an actor with a positive expression (i.e., happiness) in blocks with relevant and specific (disgust; black line) and not specific (anger; grey line) negative facial expressions.
(1.16 MB TIF)

Figure S2 Mouths' width and surface. A) Mouths' crops (160×70 pixels) obtained from the whole-face pictures of the actors. KDEF index identifying each actor is reported on the right. The negative emotion (first column) is anger, in the upper part of the panel, and disgust, at the bottom. The positive emotion (third column) is always happiness. B) Measures of mouths' width and surface, in terms of number of pixels, are reported in the tables.
(2.19 MB TIF)

## References

[1] Darwin C (1872). The expressions of the emotions in men and animals..

[2] Ekman P, Cole J (1972). Universals and cultural differences in facial expressions of emotion.. Nebraska Symposium on Motivation 1971.

[3] Izard CE, Russell JA, Fernandez-Dols JM (1997). Emotions and facial expressions: A perspective from differential emotions theory.. The Psychology of Facial Expression.

[4] Halberstadt J, Winkielman P, Niedenthal PM, Dalle N (2009). Emotional conception: how embodied emotion concepts guide perception and facial action.. Psychol Sci.

[5] Niedenthal PM (2007). Embodying emotion.. Science.

[6] Niedenthal PM, Winkielman P, Mondillon L, Vermeulen N (2009). Embodiment of emotion concepts.. J Pers Soc Psychol.

[7] Oberman LM, Winkielman P, Ramachandran VS (2007). Face to face: blocking facial mimicry can selectively impair recognition of emotional expressions.. Soc Neurosci.

[8] Fridlund AJ (1994). Human facial expression: An evolutionary view..

[9] Fridlund AJ, Russell  JA, Fernandez-Dols JM (1997). The new ethology of human expressions.. The Psychology of Facial Expression.

[10] Mead GH (1934).

[11] Dumouchel P (1995). Emotions, essai sur le corps et le social.. Le Plessis-Robinson.

[12] Britton JC, Phan KL, Taylor SF, Welsh RC, Berridge KC (2006). Neural correlates of social and nonsocial emotions: An fMRI study.. Neuroimage.

[13] Adolphs R, Baron-Cohen S, Tranel D (2002). Impaired recognition of social emotions following amygdala damage.. J Cogn Neurosci.

[14] Gallese V (2003). The manifold nature of interpersonal relations: The quest for a common mechanism.. Phil Trans Royal Soc London.

[15] Gallese V, Keysers C, Rizzolatti G (2004). A unifying view of the basis of social cognition.. Trends in Cogn Sci.

[16] Gallese V (2007). Before and below ‘theory of mind’: Embodied simulation and the neural correlates of social cognition.. Philos Trans R Soc Lond B Biol Sci.

[17] Hajcak G, Molnar C, George MS, Bolge Kr, Koola J (2006). Emotion facilitates action: a transcranial magnetic stimulation study of motor cortex excitability during picture viewing.. Psychophysiology.

[18] Oliveri M, Babiloni C, Filippi MM, Caltagirone C, Babiloni F (2003). Influence of the supplementary motor area on primary motor cortex excitability during movements triggered by neutral or emotionally unpleasant visual cues.. Exp Brain Res.

[19] Cacioppo JT, Priester JR, Berntson GG (1993). Rudimentary determinants of attitudes. II: Arm flexion and extension have differential effects on attitudes.. J Pers Soc Psychol.

[20] Förster J, Strack F (1997). Motor actions in retrieval of valenced information: a motor congruence effect.. Percept Mot Skills.

[21] Chen M, Bargh JA (1999). Nonconscious approach and avoidance behavioral consequences of the automatic evaluation effect.. Personality and Social Psychology Bulletin.

[22] Freina L, Baroni G, Borghi AM, Nicoletti R (2009). Emotive concept nouns and motor responses: attraction or repulsion?. Mem Cognit.

[23] van Dantzig S, Pecher D, Zwaan RA (2008). Approach and avoidance as action effects.. Q J Exp Psychol (Colchester).

[24] Becchio C, Sartori L, Bulgheroni M, Castiello U (2008). The case of Dr. Jekyll and Mr. Hyde: a kinematic study on social intention.. Conscious Cogn.

[25] Becchio C, Sartori L, Bulgheroni M, Castiello U (2008). Both your intention and mine are reflected in the kinematics of my reach-to-grasp movement.. Cognition.

[26] Seidel EM, Habel U, Kirschner M, Gur RC, Derntl B (2010). The impact of facial emotional expressions on behavioral tendencies in women and men.. J Exp Psychol Hum Percept Perform.

[27] Ferri F, Campione GC, Dalla Volta R, Gianelli C, Gentilucci M (2010). To me or to you? When the self is advantaged.. Exp Brain Res.

[28] Marsh AA, Ambady N, Kleck RE (2005). The effects of fear and anger facial expressions on approach- and avoidance- related behaviors.. Emotion.

[29] Horstmann G (2003). What do facial expressions convey: feeling states, behavioral intentions, or action requests?. Emotion.

[30] Repacholi BM, Gopnik A (1997). Early reasoning about desires: evidence from 14- and 18-month-olds.. Dev Psychol.

[31] Fichtenholtz HM, Dean HL, Dillon DG, Yamasaki H, McCarthy G (2004). Emotion-attention network interactions during a visual oddball task.. Brain Res Cogn Brain Res.

[32] Rotteveel M, Phaf RH (2004). Automatic affective evaluation does not automatically predispose for arm flexion and extension.. Emotion.

[33] Heuer K, Rinck M, Becker ES (2007). Avoidance of emotional facial expressions in social anxiety: the Approach-Avoidance task.. Behav Res Ther.

[34] Elliot AJ, Covington MV (2001). Approach and avoidance motivation.. Educational psychology Review.

[35] Wilkowski BM, Meier BP (2010). Bring it on: angry facial expression potentiate approach-motivated motor behavior.. J Pers Soc Psychol.

[36] DeBruine LM (2005). Trustworthy but not lust-worthy: context-specific effects of facial resemblance.. Proc Biol Sci.

[37] Freeman JB, Ambady N (2009). Motions of the hand expose the partial and parallel activation of stereotypes.. Psychol Sci.

[38] Goeleven E (2000). The Karolinska Directed Emotional Faces: A validation study.. Cognition & Emotion.

[39] Schneider W, Eschman A, Zuccolotto A (2002). E-Prime user's guide..

[40] Schneider W, Eschman A, Zuccolotto A (2002). E-Prime reference guide..

[41] Bonino S, Coco AL, Tani F (1998).

[42] Davis M (1980). A multidimensional approach to individual differences in empathy.. JSAS Cat Sel Doc Psycho.

[43] Davis M (1983). Measuring individual differences in empathy: evidence for a multidimensional approach.. J Pers Soc Psychol.

[44] Davis M (1996). Empathy: A Social Psychological Approach..

[45] Benjamini Y, Hochberg Y (1995). Controlling the false discovery rate: a practical and powerful approach to multiple testing.. J Roy Statist Soc Ser B.

[46] Fitts PM (1954). The information capacity of the human motor system in controlling the amplitude of movement.. J Exp Psychol.

[47] Wicker B, Keysers C, Plailly J, Royet JP, Gallese V (2003). Both of us disgusted in my insula: the common neural basis of seeing and feeling disgust.. Neuron.

[48] Jabbi M, Bastiaansen J, Keysers C (2008). A common anterior insula representation of disgust observation, experience and imagination shows divergent functional connectivity pathways.. PLoS One.

[49] Caruana F, Jezzini A, Sbriscia Fioretti B, Stoianov I, Umiltà MA (2008). Intracortical Microstimulation Mapping of the Inner Perisylvian Regions: Effects on Behavior and ECG outcome..

[50] Aviezer H, Hassin RR, Ryan J, Grady C, Susskind J (2008). Angry, disgusted, or afraid? Studies on the malleability of emotion perception.. Psychol Sci.

[51] Leslie KR, Johnson-Frey SH, Grafton ST (2003). Functional imaging of face and hand imitation: towards a motor theory of empathy.. Neuroimage.

[52] Hatfield E, Cacioppo JT, Rapson RL (1993). Emotional contagion.. Current Directions in Psychol Sci.

