# Supplementary figures and images for: When Action Meets Emotions: How Facial Displays of Emotion Influence Goal-Related Behavior
Source: PLoS One. 2010 Oct 1;5(10):e13126. doi: 10.1371/journal.pone.0013126 (PMC2948517; doi:10.1371/journal.pone.0013126)

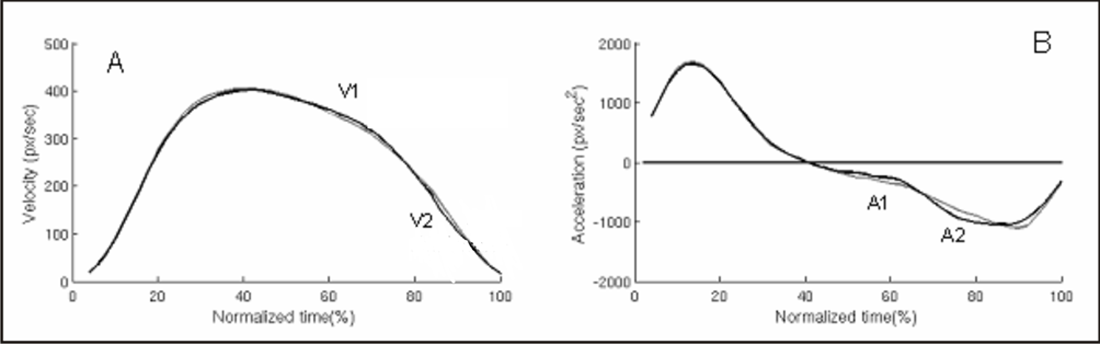

Supplement: Figure S1 — Velocity and acceleration profiles. Absolute time-normalized profiles of velocity (A) and acceleration (B) during feeding an actor with a positive expression (i.e., happiness) in blocks with relevant and specific (disgust; black line) and not specific (anger; grey line) negative facial expressions. (1.16 MB TIF) [file pone.0013126.s001.tif]

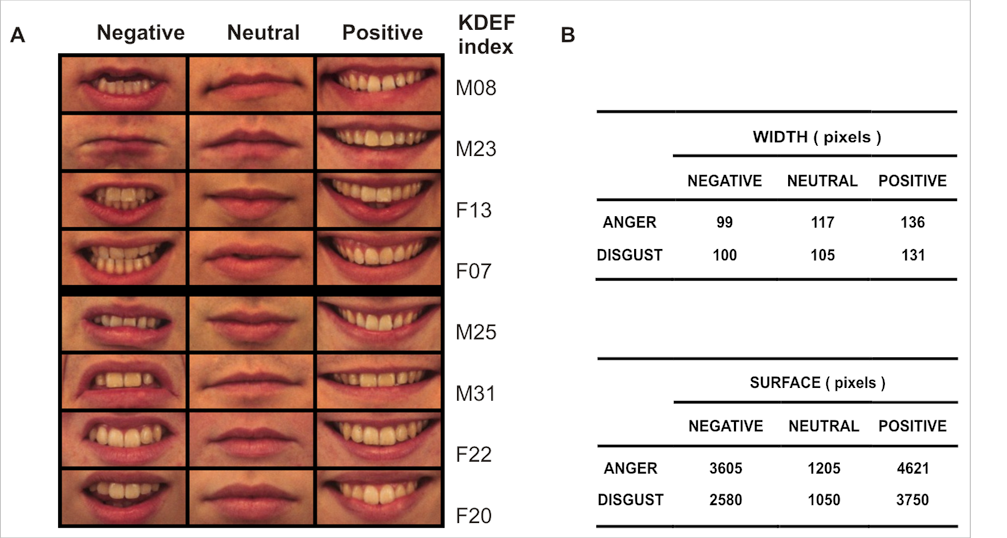

Supplement: Figure S2 — Mouths' width and surface. A) Mouths' crops (160×70 pixels) obtained from the whole-face pictures of the actors. KDEF index identifying each actor is reported on the right. The negative emotion (first column) is anger, in the upper part of the panel, and disgust, at the bottom. The positive emotion (third column) is always happiness. B) Measures of mouths' width and surface, in terms of number of pixels, are reported in the tables. (2.19 MB TIF) [file pone.0013126.s002.tif]
